# Vitrimerization of Crosslinked Unsaturated Polyester Resins: A Mechanochemical Approach to Recycle and Reprocess Thermosets

**DOI:** 10.1002/gch2.202200036

**Published:** 2022-04-28

**Authors:** Alireza Bandegi, Mehrad Amirkhosravi, Haotian Meng, Mir Karim Razavi Aghjeh, Ica Manas‐Zloczower

**Affiliations:** ^1^ Department of Macromolecular Science and Engineering Case Western Reserve University 2100 Adelbert Road, Kent Hale Smith Bldg Cleveland OH 44106 USA; ^2^ Institute of Polymeric Materials Faculty of Polymer Engineering Sahand University of Technology Sahand New Town Tabriz 51335–1996 Iran

**Keywords:** recycling, thermoset waste, transesterification reaction, unsaturated polyester resins, vitrimerization

## Abstract

Unsaturated polyester resins (UPRs) are expansively used in different applications and recycling the significant amounts of UPR waste is still a universal problem. Vitrimerization is a feasible, environmental‐friendly, cost effective, and operative method, which can be used for recycling the crosslinked UPRs. In this method, the thermoset permanent network is changed into a dynamic network similar to the vitrimer‐type polymers. The results show that the existence of a transesterification catalyst in the system significantly enhances the efficiency of vitrimerization. The vitrimerized UPR thermosets can be reprocessed three times with mechanical properties comparable to the initial UPR. The results show that the excess of external hydroxyl groups in the system can prevent the formation of zinc ligand complexes in the network and consequently reduce the crosslinked density and mechanical properties of vitrimerized samples. The vitrimerized thermoset powder can be reprocessed through injection molding, extrusion, and compression molding which are conventional thermoplastic processing techniques. The unrecyclable UPR thermoset wastes can be recycled and reused through vitrimerization with the least loss in mechanical properties.

## Graphical abstract

Thermoset unsaturated polyester resin waste can be recycled through a mechanochemical approach based on ball milling the ground waste with an appropriate transesterification catalyst followed by compression molding, extrusion, or injection molding. This vitrimerization approach converts the permanent network into a vitrimer‐dynamic network, which can be recycled multiple times without significant loss in mechanical properties.

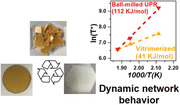

## 1 Introduction

Unsaturated polyester resins (UPRs) as the main precursors of the thermoset polyesters, are one of the main categories of polymer resins used around the world, due to their versatility and low cost.^[^ ^1^ ^]^ UPRs are extensively used in the production of thermoset polymer composites which can be processed at different temperatures.^[^ ^2^ ^]^ Furthermore, the ability to adjust different properties of UPRs such as corrosion resistance, mechanical properties, wetting properties with different additives, as well as their processability, makes them the material of choice for a broad range of applications.^[^ ^1^ ^]^ In addition, the 3D network structure of crosslinked UPR render them thermally and chemically stable. On the other hand, the waste from UPRs and their composites during manufacturing and end‐of‐life components are extensively accumulating.^[^ ^1^ ^]^ Furthermore, the existence of high concentration of hazardous pollutants such as styrene monomer in the UPRs leaves harmful imprints on the environment. Therefore, it is crucial to design an operative and efficient method for recycling crosslinked UPR wastes. So far, different recycling methods, such as mechanical processes alike grinding,^[^ ^3^, ^4^, ^5^ ^]^ solvolysis,^[^ ^6^, ^7^ ^]^ fluidized‐bed process,^[^ ^3^, ^4^ ^]^ and glycolysis have been explored and practiced.^[^ ^8^ ^]^ However, the use of toxic chemicals and harsh treatment conditions reduce the efficiency of these methods. In addition, many byproducts produced by these methods are harmful to the environment. Mechanical recycling encompasses crushing the composite resin followed by fine grinding to smaller fragments with size of 50–100 mm.^[^ ^3^ ^]^ However, loading of these recyclates as fillers above 10% reduces the mechanical properties and impedes processing due to the increased viscosity of the molding compound.^[^ ^3^ ^]^ In addition, to the extent of our knowledge, no techniques have been practiced to make new parts directly from waste thermosets as is the case with thermoplastic materials.

The dynamic covalent chemistries have attracted significant interest since Leibler et al.^[^ ^9^ ^]^ applied the concept of vitrimer on epoxy networks. The advantage of dynamic covalent networks in vitrimer polymers compared to traditional thermoset polymers is to maintain the integrity of the network at high temperatures while the topology rearrangement occurs.^[^ ^10^, ^11^, ^12^, ^13^ ^]^ Vitrimers can be reprocessed, healed, and therefore recycled through topology rearrangement. Different types of vitrimer networks such as epoxy acids,^[^ ^14^, ^15^ ^]^ siloxanes,^[^ ^16^ ^]^ polyolefins,^[^ ^17^ ^]^ polyureas,^[^ ^18^ ^]^ and polyimines^[^ ^19^ ^]^ have been explored. However, none of these systems have been commercialized yet to replace thermosets.

The concept of vitrimerization has been recently developed in our group to obtain vitrimer‐type dynamic networks from permanently cross‐linked thermosets through a simple one‐step process.^[^ ^20^, ^21^, ^22^, ^23^, ^24^ ^]^ In this method, the exchangeable metal−ligand sites form via ball milling the thermoset network with a proper catalyst.^[^ ^20^, ^21^ ^]^ The properties of the initial thermosets are mostly recovered in the vitrimerized systems. The vitrimerization process enables a practical, cost effective, ecofriendly, and commercially scalable approach to overcome the challenges of thermoset recycling.

In this study, the transesterification reaction^[^ ^25^, ^26^, ^27^, ^28^, ^29^, ^30^, ^31^ ^]^ is utilized for vitrimerization of the styrene‐assisted crosslinked UPR. Thermoset polyester materials, containing freely available hydroxyl groups can be converted into vitrimers using a proper transesterification catalyst. Besides the catalyst, crosslink exchange can be accelerated by increasing the amount of free hydroxyl groups (different alcohols)^[^ ^25^ ^]^ leading even to catalyst‐free vitrimers.^[^ ^32^ ^]^ In our previous work, we investigated the effect of adding cellulose nanocrystals as feed stock of free hydroxyl groups on the recycling efficiency of epoxy systems. The results showed that the cellulose nanocrystals can both facilitate the transesterification reaction and also improve the thermomechanical properties of the vitrimerized epoxy acting as nanofillers.^[^ ^24^ ^]^ Herein, we also explored the effect of adding external hydroxyl groups (dipentaerythritol powder) besides the catalyst on the efficacy of UPR vitrimerization. This work provides a platform to design a flexible approach for recycling UPR thermoset wastes to high value‐added product.

## 2 Results and Discussion

Vitrimerized UPRs were obtained through ball milling of the crosslinked unsaturated polyester with different concentrations of alcohol (10 and 15 wt% relative to UPR) and catalyst (10 mol% relative to hydroxyl groups in alcohol) (**Table** **1**). The crosslinked UPR is also ball milled without addition of alcohol or catalyst to explore the effect of ball milling on the formation of radicals in the UPR network.

**Table 1:** Initial composition of the vitrimerized samples and reference material

Sample code	Unsaturated polyester [g]	Dipentaerythritol [g]	Catalyst [g] (10 mol% relative to hydroxyl groups in dipentaerythritol)
Ball‐milled UPR	10	0	0
V‐1	10	1.5	0.649
V‐2	10	1.0	0.433
V‐3	10	0	0.649^a)^

^a)^ The sample V‐3 is prepared with the same amount of catalyst as sample V‐1.

**Figure 1:**
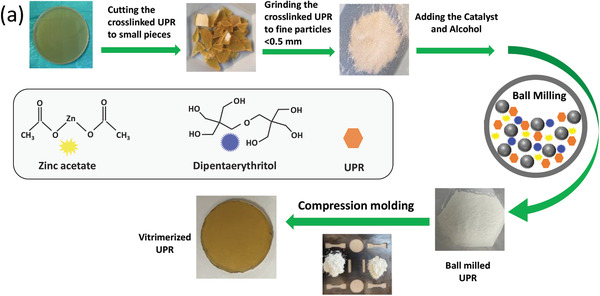
a) Vitrimerization of UPR thermoset.

The incorporation of an appropriate catalyst facilitates the transesterification exchange reaction, and the crosslinked thermoset network converts to a dynamic network. Zinc acetate was selected as catalyst for the transesterification reaction because it is nontoxic and highly efficient.^[^ ^31^, ^33^, ^34^ ^]^ The metal–ion interactions in these networks have been comprehensively investigated.^[^ ^35^, ^36^ ^]^ In a typical polyester vitrimer network, carboxylate groups attach as a ligand to Zn^2+^.^[^ ^31^, ^33^ ^]^ These complexes are linkages for the transesterification exchange reaction and serve also as exchangeable unstable crosslink junctions in the vitrimer network. **Figure** **1** shows the vitrimerization process in this work.

**Figure 2:**
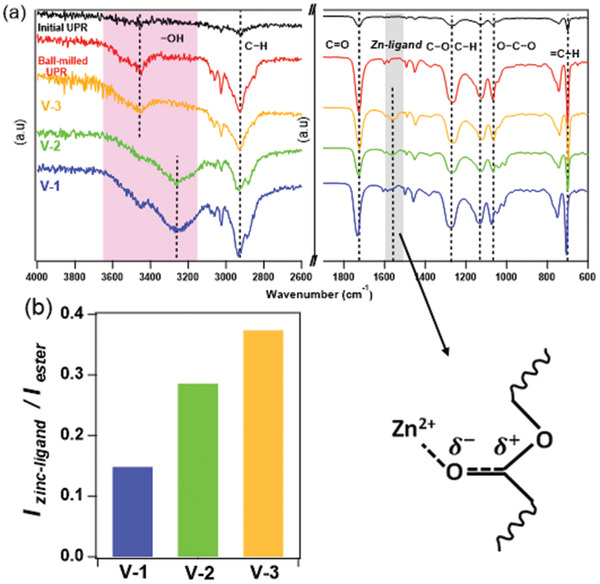
a) FTIR spectra of initial UPR and vitrimerized samples and b) FTIR peak intensity of zinc‐carboxylate complex (1560−1520 cm^−1^) to ester (1760−1690) cm^−1^ in vitrimerized samples.

The Fourier transform infrared spectroscopy (FTIR) results, **Figure** **2**a, show that there are enough functional groups of esters (1760−1690 cm^−1^) and hydroxyls (3600−3200 cm^−1^) in the commercial UPR to reprocess this material through transesterification reaction. The existence of hydroxyl groups in the UPR network has been reported in the literature.^[^ ^37^, ^38^, ^39^ ^]^ However, it is crucial to investigate the effect of catalyst and alcohol on the efficacy of the transesterification exchange reaction to enable applying the process to a wide range of polyester thermosets with different formulations and degrees of crosslinking. By adding the catalyst and alcohol, the peaks related to carboxylate‐zinc vibrations appear in the spectrum (1560−1520 cm^−1^) and intensity of the peaks corresponding to hydroxyl groups increases upon ball milling. It is observed that the intensity of the peaks related to carboxylate‐zinc vibrations follows the order of V‐3 > V‐2 > V‐1 (Figure 2b), even though there is less catalyst content in the V‐2 sample compared to V‐1. These results seem to indicate that adding excess of external alcohol in the thermoset matrix can inhibit the formation of zinc carboxylate complexes during the ball milling.

**Figure 3:**
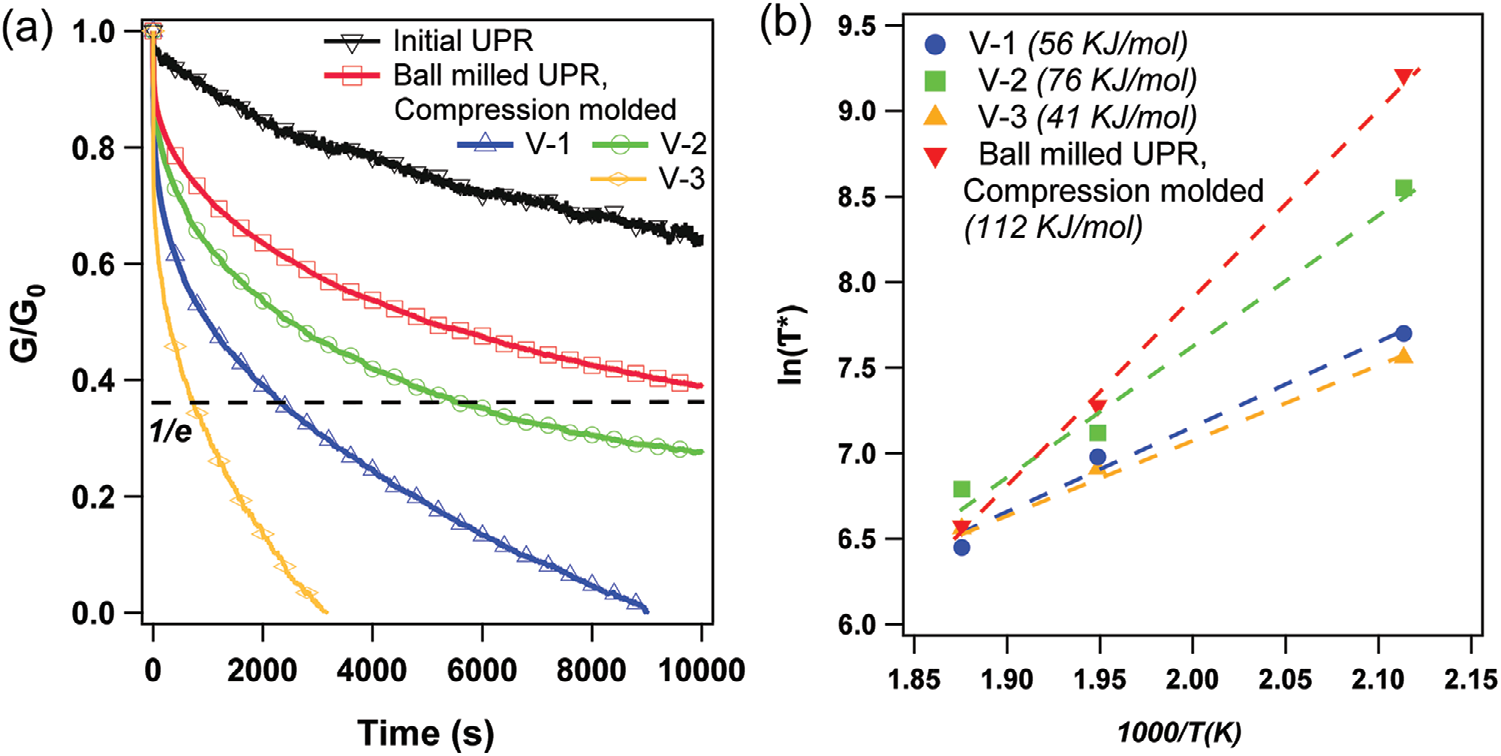
a) Stress relaxation curves of initial UPR and vitrimerized samples at 200 °C and b) Arrhenius plot of the measured relaxation times at various temperatures obtained from the data presented in Figure S1 in the Supporting Information.

**Figure** **3**a shows the stress relaxation results for different samples at 200 °C. The results are similar to other vitrimer polymers in which the time and temperature‐dependent stress relaxation behavior can be expressed by an Arrhenius equation (Figure S1, Supporting Information).

Since the permanently cross‐linked networks cannot relax the stress, these results point out to the exchange reaction and consequently topology rearrangement in the vitrimerized network. As expected, the V‐3 samples have the fastest stress relaxation rate, due to the enhanced formation of metal–ligand complexes in the network.

Moreover, the results show that the concentration of hydroxyl groups in the commercial UPR resin studied in this work after ball milling is sufficient to enable reprocessing of the ball‐milled UPR without catalyst (Figure 2a). However, reprocessing of the ball‐milled UPR without catalyst through conventional methods such as injection molding is not efficient due to the limited transesterification reaction rate (Figure 3a).

The activation energy (*E*_a_) for the transesterification reaction can be obtained from the Arrhenius equation τ*(*T*) = τ*_0_(*T*) exp (*E*_a_/*RT*). The relaxation time τ* is the time where the stress is reduced to 37% of its initial value. The activation energies for vitrimerized samples (Figure 3b) follow the order of V‐3 < V‐1 < V‐2 < ball‐milled UPR. The lowest activation energies are for the V‐3 and V‐1 samples with the highest amount of catalyst in the system (Table 1). On the other hand, the activation energy for the ball‐milled UPR without catalyst is significantly higher compared to the other samples. The high activation energy for ball‐milled UPR indicates the importance of adding catalyst in the vitrimerization process to facilitate the transesterification reaction.

The dilatometry test is used to measure the topology freezing transition temperature, *T*_v_, of vitrimerized samples and the results are shown in Figure S2 in the Supporting Information. The *T*_v_ for all the vitrimerized samples is around 180 °C. The thermogravimetric analysis (TGA) results in Figure S3 in the Supporting Information show that the initial UPR and vitrimerized samples are stable up to 260 °C.

**Figure 4:**
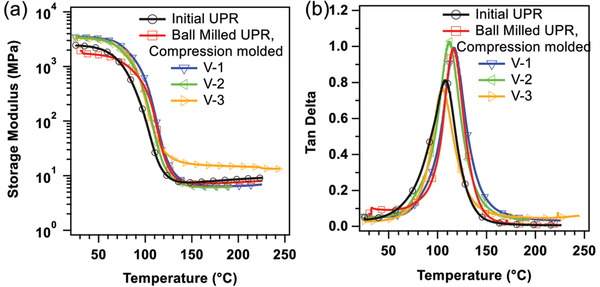
a) The storage modulus and b) tan delta of initial crosslinked UPR and vitrimerized samples.

The dynamic mechanical thermal behavior of vitrimerized samples is investigated and compared with the initial UPR. **Figure** **4** presents the storage modulus *E*′ and tan (δ) of the initial cured unsaturated polyester and vitrimerized samples as a function of temperature. The storage modulus of the vitrimerized samples is higher than the initial polyester at room temperature. The storage modulus starts to decrease linearly before the glass transition temperature (*T*_g_) and then sharply decreases around *T*_g_ reaching a rubbery plateau. The molecular weight between successive crosslinks, *M*_e_, can be obtained from *G*_p_ ≅ *ρRT/M*_e_, where *G*_p_ is the storage modulus of the rubbery plateau at temperature *T* and ρ is the polymer density.^[^ ^40^, ^41^, ^42^ ^]^ A higher rubbery plateau modulus points out to higher crosslinking density. Thus, from the results we can conclude that the V‐3 sample has the highest crosslinking degree. These results show that the zinc carboxylate complexes created through vitrimerization act as physical crosslinking junctions and increasing the catalyst amount (i.e., increasing zinc carboxylate complexes) results in higher crosslinking density and storage modulus. This result strengthens our conclusion from the FTIR results in which the excess addition of external hydroxyl groups can inhibit the formation of zinc carboxylate complexes, and consequently reduce the crosslinking density. As shown in Figure 4b, the *T*_g_ of vitrimerized samples are comparable with the initial UPR.

The tensile and impact strength properties of the initial and vitrimerized samples are displayed in **Table** **2**. Overall, the ball‐milled UPR and the vitrimerized samples show a more rigid network with lower elongation at break. The vitrimerized sample with the highest content of dipentaerythritol (V‐1) has the lowest strength and elongation at break. The excess alcohol present in the system may contribute to the dynamic network heterogeneity and may result in overall poorer mechanical properties. The vitrimerized sample V‐2 was recycled three times without addition of catalyst or alcohol and the mechanical properties were well preserved. The morphology of the initial and vitrimerized UPR was characterized by scanning electron microscopy and the images are shown in Figure S4 in the Supporting Information. The initial UPR exhibits a relatively smooth surface, whereas the vitrimerized samples, in particular V‐1 show rough surfaces in agreement with the tensile test results.

**Table 2:** Mechanical properties of initial UPR and vitrimerized samples

Sample code	Young's modulus [GPa]	Tensile strength [MPa]	Elongation at break [%]	Impact strength [kJ m^−2^]
Initial UPR	1.7 ± 0.1	18.2 ± 3.3	4.1 ± 0.1	1.47 ± 0.02
Ball‐milled UPR	1.2 ± 0.2	18.6 ± 1.6	3.6 ± 0.3	1.33 ± 0.02
V‐1	0.9 ± 0.3	14.8 ± 1.7	2.4 ± 0.0	1.17 ± 0.09
V‐2	1.9 ± 0.1	20.8 ± 0.5	2.8 ± 0.5	1.30 ± 0.06
V‐3	1.8 ± 0.4	21.3 ± 1.1	3.3 ± 0.0	1.07 ± 0.04
V‐2 reprocessed (*×* 2)	2.3 ± 0.3	20.0 ± 1.5	3.3 ± 0.3	1.40 ± 0.05
V‐2 reprocessed (× 3)^a)^	2.2	21.0	3.0	0.95

^a)^ One experiment was performed for this sample; thus, the result does not have standard deviation.

**Figure 5:**
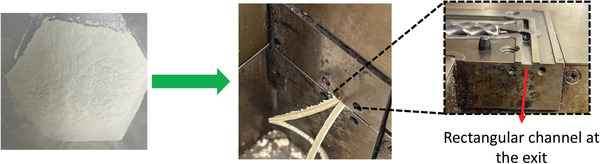
Vitrimerized UPR powder extruded to strands at 200 °C.

We also explored the vitrimerized samples processability in extrusion. The initial UPR and the ball‐milled UPR could not be extruded. By contrast, the vitrimerized sample V‐3 with the highest crosslinking density was extruded using a twin‐screw counter rotating minilab operating at a temperature of 200 °C and screw speed of 50 rpm with 10 min residence time. In addition, some of the extruded strands were forced into a rectangular shaped mold, a process similar to injection molding (**Figure** **5**).

The work presented here is an exploratory study and no process optimization was attempted. Further optimization will allow tailoring the vitrimerization process to specific UPR thermoset waste and better control of the recycled material properties.

## 3 Conclusion

The concept of vitrimerization has been applied on a crosslinked UPR thermoset. In this study, the activation energy and welding efficacy were investigated for vitrimerization of UPR thermosets with different compositions. Stress relaxation experiments confirm that the unrecyclable thermoset was successfully converted into a vitrimer. The intractable 3D network of the initial UPR does not allow the stress to relax. By contrast, the vitrimerized samples relax the stress quickly due to the transesterification reaction.

It was found that the amount of catalyst and formation of zinc carboxylate complexes are more important for converting the UPR thermoset into a vitrimer than the concentration of external hydroxyl groups in the system. This method shows potential for converting UPR thermoset waste already present in the market into vitrimers and is appropriate for industrial applications. The results can provide guidance to overcome the challenges in recycling unsaturated polyester thermoset polymers and tailor the properties of the vitrimerized systems with the least environmental impact.

## 4 Experimental Section

### Materials

Commercial unsaturated polyester resin, 77 polyester molding resin, and methyl ethyl ketone peroxide (MEKP) as initiator were purchased from FIBREGLAST. Dipentaerythritol and zinc acetate were purchased from Sigma‐Aldrich.

### Preparation of Crosslinked UPR

Polyester molding resin (50 g) and MEKP (0.625 g) were mixed at room temperature for 1 min. Then the resin was crosslinked in a Teflon mold for 24 h at room temperature and for further 3 h at 80 °C, the later stage was under vacuum.

### Vitrimerization Process

The cured UPRs were cut into small pieces and then grinded into fine particles (<500 µm). Then the mixture of the cured UPR fine particles, transesterification catalyst (zinc acetate), and dipentaerythritol at different concentrations was dried in an oven overnight under vacuum and then poured into the ball mill tank (Fritsch pulverisette 6), purged with N_2_, and finally ball milled for 60 min at a speed of 570 rpm into ultrafine powder mixture. The resultant ultrafine powder mixtures were compression molded at 200 °C and 5 MPa for 60 min in a mold made from stainless steel, to obtain vitrimerized samples. The same procedure was followed for additional reprocessing of the vitrimerized samples except that no catalyst and alcohol were added to the system during the ball milling.

### Characterizations

*Dynamic Mechanical Analysis*: TA Instrument Q800 was used in tensile mode to determine the dynamic mechanical properties, storage modulus (*E*′), and tan (δ) with a constant frequency of 1 Hz and a strain amplitude of 0.05%. A scanning rate of 5 °C min^−1^ from 30 to 230 °C was used during the test. The peak of tan (δ) curves was considered for determining the glass transition temperature (*T*_g_) of different samples. Dilatometry was performed in tension mode. An elongational stress of 100 kPa was used while heating the samples with rate of 5 °C min^−1^ from 50 to 220 °C. The strain was measured simultaneously during the test.^[^ ^43^ ^]^

*FTIR*: FTIR analyses were performed using an Agilent Cary 630 FTIR spectrophotometer within the wavenumber range of 4000−600 cm^−1^.

*Mechanical Testing*: The samples for tensile tests were molded with sample size of 1.5 mm × 11 mm × 60 mm and the tests were performed on an MTS Insight tensile instrument with strain rate of 5 mm min^–1^. The impact tests were performed on QPI‐IC‐12J Universal Impact Tester, according to Notched Izod Impact testing method, ASTM D256. In mechanical testing, each sample was tested multiple times to confirm reproducibility.

*Rheology*: Stress relaxation experiments were performed on a TA ARES‐G2 rheometer with a 25 mm parallel plate geometry. The average thickness of the samples was 1.5 mm. The samples were equilibrated for 20 min at desired temperature and then a 1% step strain was applied. A normal force of 10 N was maintained during the experiment to avoid the gap between the sample and plate.

*TGA*: The thermal stability of initial UPR and vitrimerized samples was explored by TGA (TA Instruments Q500). The samples were about 10 mg each in an aluminium pan and heated from room temperature to 600 °C with rate of 10 °C min^−1^ under a constant N_2_ flow.

## Conflict of Interest

The authors declare no conflict of interest.

## Supporting information

Supporting Information

## Data Availability

The data that support the findings of this study are available from the corresponding author upon reasonable request.
