# Supplementary material for: Vitrimerization of Crosslinked Unsaturated Polyester Resins: A Mechanochemical Approach to Recycle and Reprocess Thermosets
Source: Glob Chall. 2022 Apr 28;6(7):2200036. doi: 10.1002/gch2.202200036 (PMC9284659; doi:10.1002/gch2.202200036)
Supplement: Supplementary file 1 — Supporting Information [file GCH2-6-2200036-s001.pdf]

Global Challenges
Open Access

Supporting Information

for *Global Challenges,* DOI: 10.1002/gch2.202200036

Vitrimerization of Crosslinked Unsaturated Polyester Resins: A Mechanochemical Approach to Recycle and Reprocess Thermosets

*Alireza Bandegi, Mehrad Amirkhosravi, Haotian Meng, Mir Karim Razavi Aghjeh, and Ica Manas-Zloczower**

Supporting information

Vitrimerization of Crosslinked Unsaturated Polyester Resins: A Mechanochemical Approach to Recycle and Reprocess Thermosets

Alireza Bandegi[1], Mehrad Amirkhosravi[1], Haotian Meng[1], Mir Karim Razavi-Aghjeh[1,2], Ica Manas-Zloczower[1*]

[*] Corresponding author. Email: ixm@case.edu.

[1]Department of Macromolecular Science and Engineering, Case Western Reserve University, 2100 Adelbert Road, Kent Hale Smith Bldg, Cleveland OH. 44106, USA

[2]Institute of Polymeric Materials, Faculty of Polymer Engineering, Sahand University of Technology, Sahand New Town, Tabriz, Iran P.C: 51335-1996

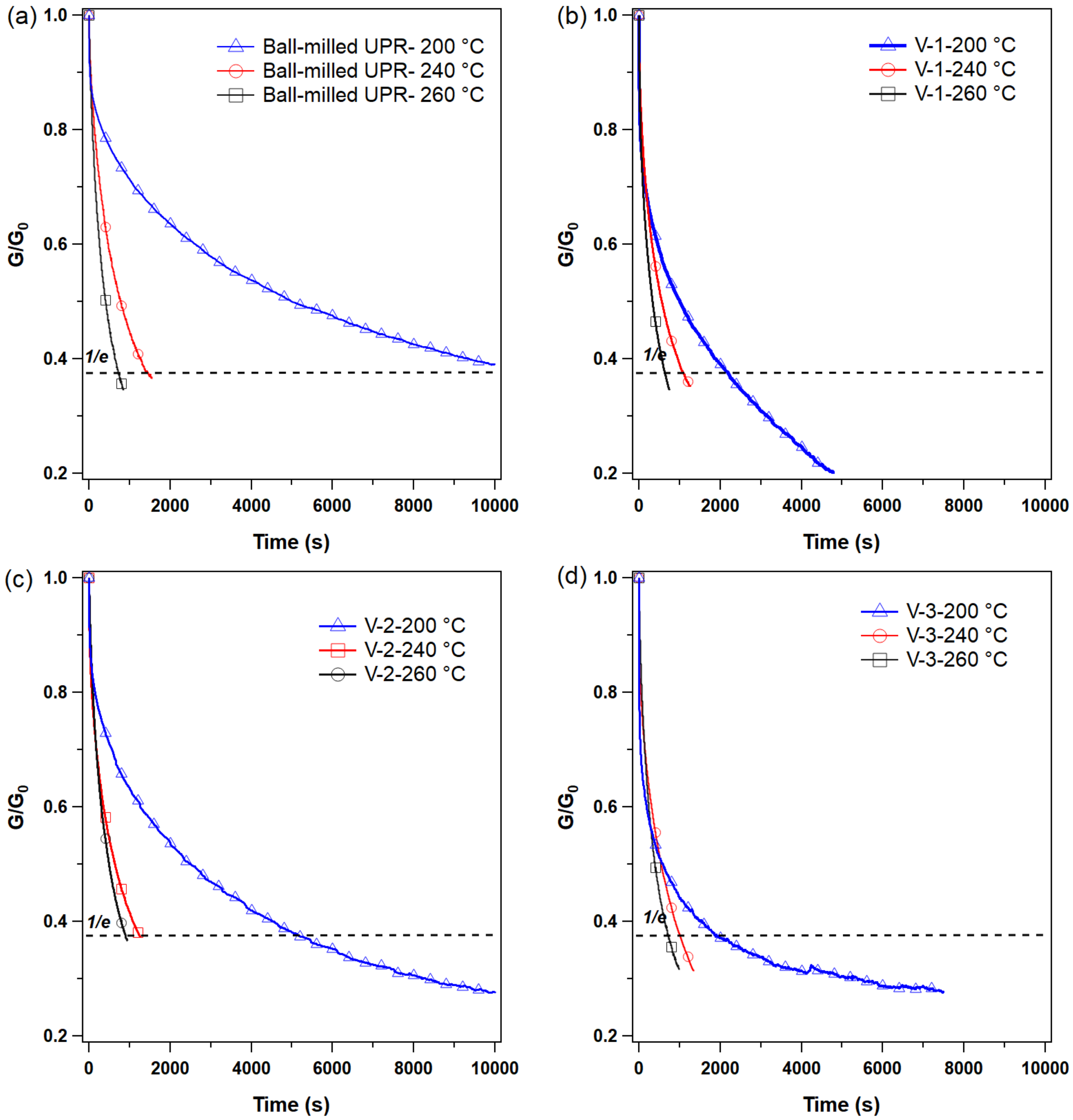

Figure S1. Stress relaxation curves of ball milled and vitrimerized samples at different temperatures.

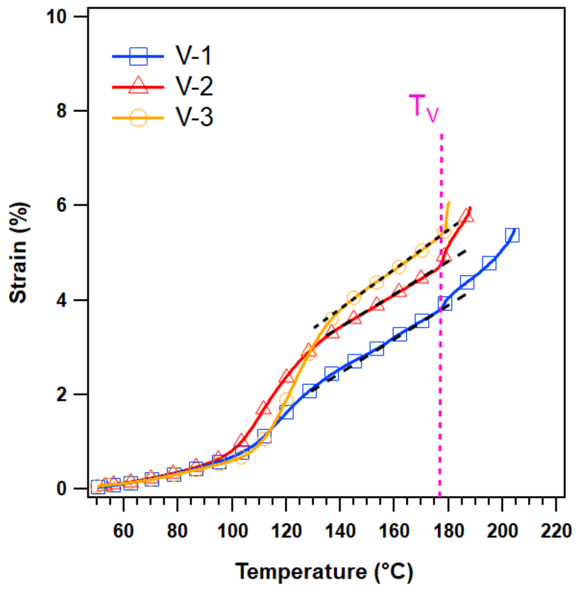

Figure S2. Dilatometry results for vitrimerized samples.

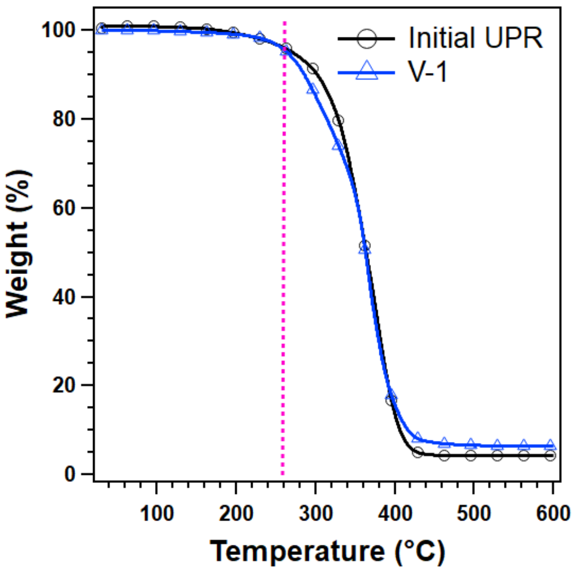

Figure S3. TGA curves of weight loss for initial UPR and vitrimerized sample.

The cross-section of the samples were observed using Apreo 2 scanning electron microscopy.

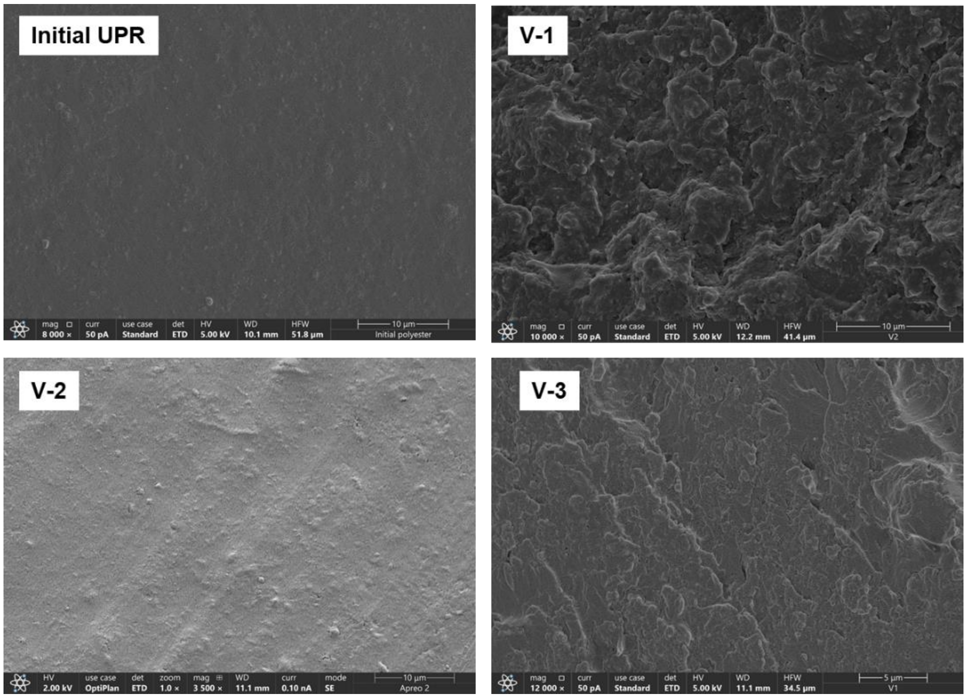

Figure S4. SEM images of cross sections for the initial and vitrimerized UPR.
